# Parliament and the Profession

**Published:** 1918-03-09

**Authors:** 


### PARLIAMENT AND THE PROFESSION.

### Discharged Soldiers: Hospital Treatment.

Sir A. Griffith-Boscawen informed Colonel Ashley that at his instance the Army Council have issued instructions to the London and other commands to submit proposals without delay for obtaining accommodation for serving soldiers elsewhere than in civil hospitals, so that the immediate requirements for discharged men may be met. At the same time the Army Council have agreed to admit a limited number of discharged men to the military orthopædic hospitals for in-patient treatment, and to accept patients in the military hospitals for facial injuries.

### Promotions in the Army Medical Service.

Mr. Macpherson informed Mr. Llewelyn Williams that recent promotions in the Army Medical Service have been made in some instances among officers who perform purely professional duties, and in others both professional and administrative capacity were duly considered. The promotions were made specially for the purposes of the war, and officers not so selected are still eligible for promotion in the ordinary course. The recommendations of General Officers Commanding were obtained and carefully considered, and the selections were made by the Adjutant-General, in consultation with the Director-General, Army Medical Service, after exhaustive inquiry, and were finally submitted to the Secretary of State for approval.

### Surgical Boots for Wounded Soldiers.

Sir A. Griffith-Boscawen informed Colonel Ashley that it is the practice of the Ministry of Pensions to provide two pairs of surgical boots when required, and to withhold the issue of the second pair for a short time to enable it to be seen whether the style of boot provided is satisfactory. With regard to surgical appliances generally arrangements are made to effect repairs and renewals as and when they become necessary.

### Extra Rations for Invalids.

In reply to questions directed to the Ministry of Food, Mr. Clynes stated that instructions have been issued to Food Offices in the London and Home Counties area to provide for an extra ration in the case of those who are suffering from tuberculosis and diabetes, and are certified by their medical attendants that they require extra or special food. The provision applies to discharged soldiers and to patients in sanatoria in common with other civilians. (See col. 2, "Food Problem Notes," p. 492.)

